# Supplementary material for: NEK1-mediated retromer trafficking promotes blood–brain barrier integrity by regulating glucose metabolism and RIPK1 activation
Source: Nat Commun. 2021 Aug 10;12:4826. doi: 10.1038/s41467-021-25157-7 (PMC8355301; doi:10.1038/s41467-021-25157-7)
Supplement: Supplementary file 1 — Supplementary Figures [file 41467_2021_25157_MOESM1_ESM.pptx]

## Slide 1
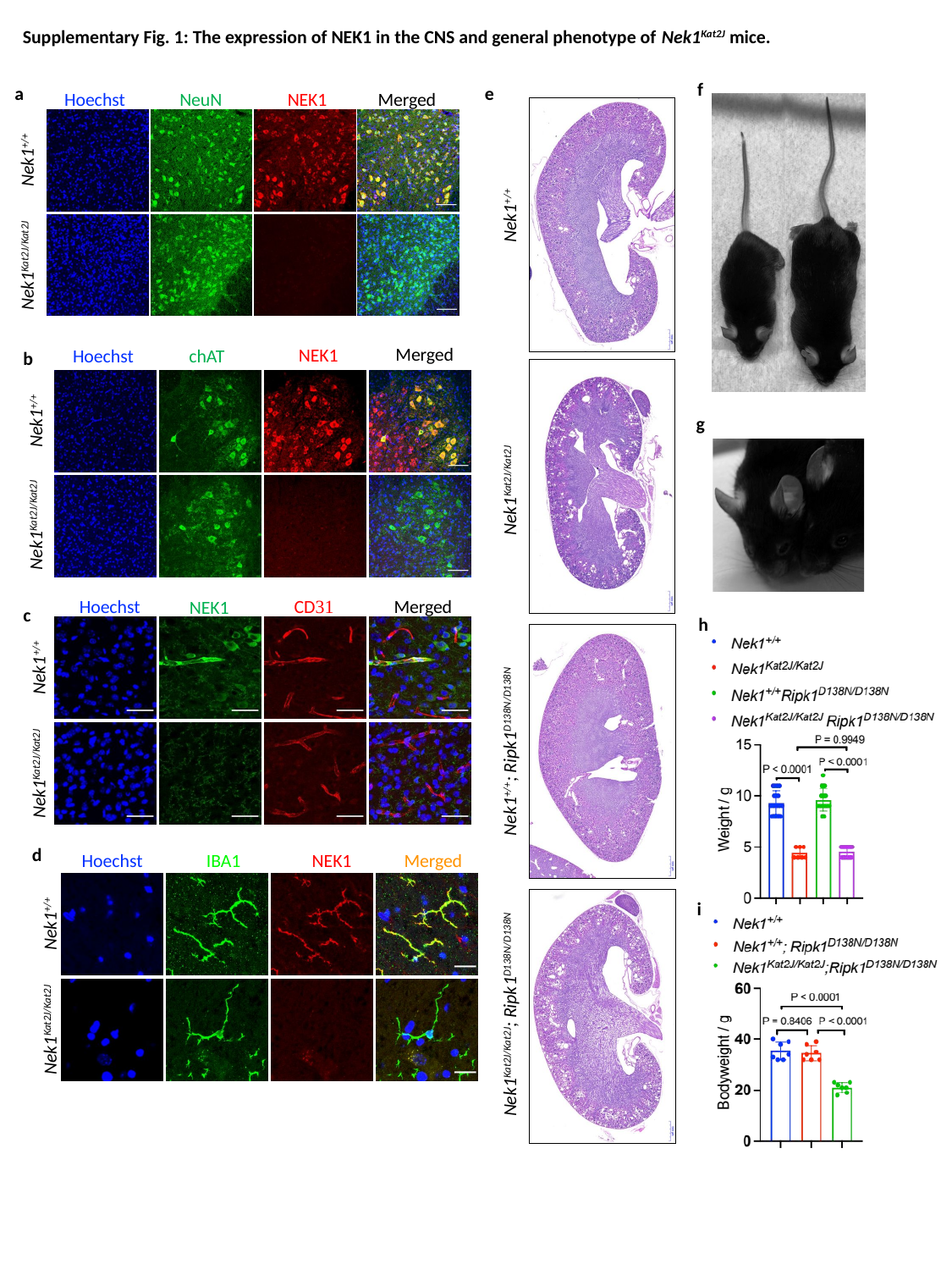

Supplementary Fig. 1: The expression of NEK1 in the CNS and general phenotype of Nek1Kat2J mice.
f
a
e
Hoechst
NeuN
NEK1
Merged
Nek1+/+
Nek1+/+
Nek1Kat2J/Kat2J
Merged
NEK1
chAT
Hoechst
b
Nek1+/+
g
Nek1Kat2J/Kat2J
Nek1Kat2J/Kat2J
Merged
Hoechst
CD31
NEK1
c
h
Nek1+/+
Nek1+/+; Ripk1D138N/D138N
Nek1Kat2J/Kat2J
d
Hoechst
IBA1
NEK1
Merged
i
Nek1+/+
Nek1Kat2J/Kat2J; Ripk1D138N/D138N
Nek1Kat2J/Kat2J

## Slide 2
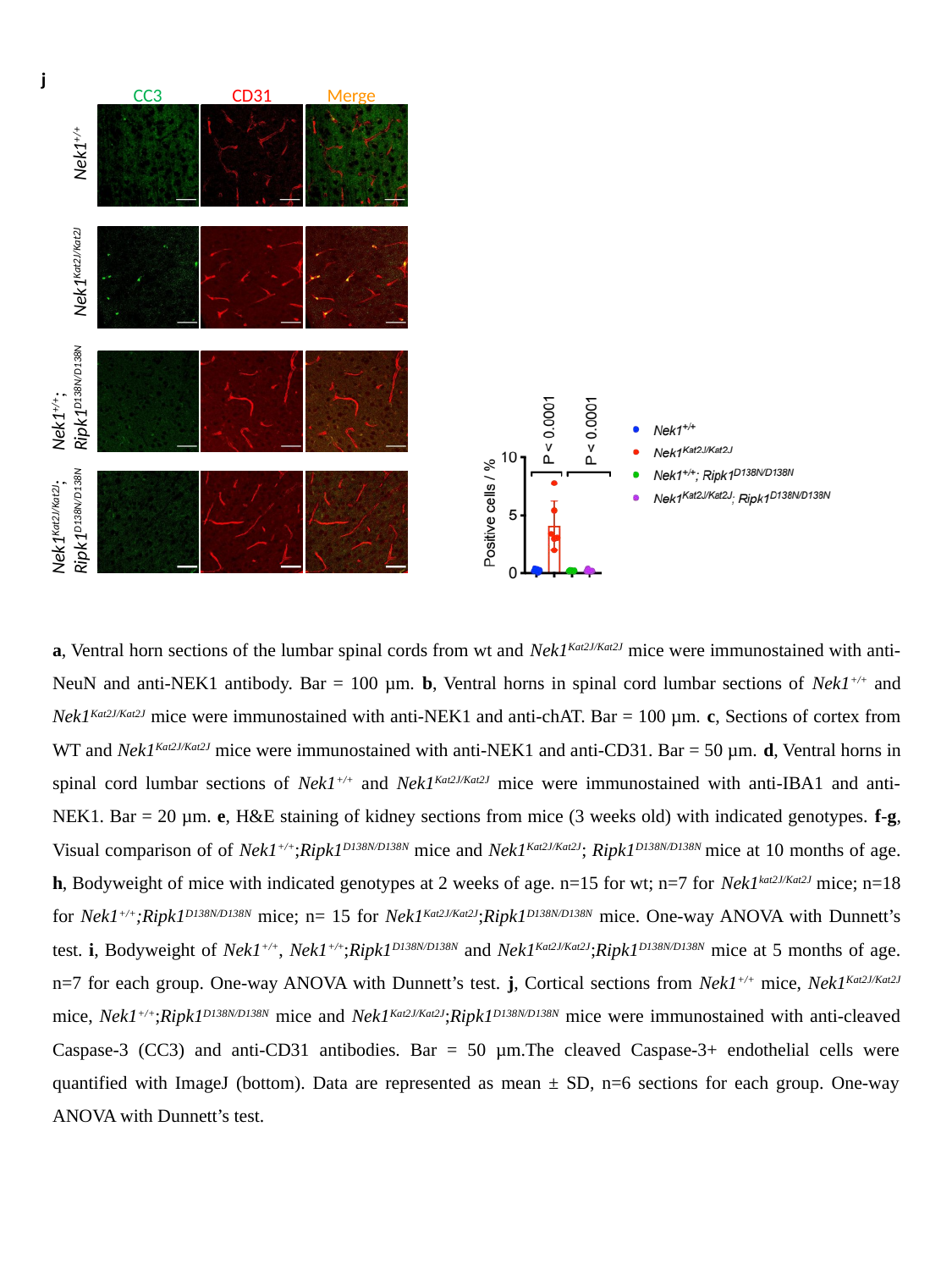

j
CC3
CD31
Merge
Nek1+/+
Nek1Kat2J/Kat2J
Nek1+/+;
Ripk1D138N/D138N
Nek1Kat2J/Kat2J;
Ripk1D138N/D138N
a, Ventral horn sections of the lumbar spinal cords from wt and Nek1Kat2J/Kat2J mice were immunostained with anti-NeuN and anti-NEK1 antibody. Bar = 100 µm. b, Ventral horns in spinal cord lumbar sections of Nek1+/+ and Nek1Kat2J/Kat2J mice were immunostained with anti-NEK1 and anti-chAT. Bar = 100 µm. c, Sections of cortex from WT and Nek1Kat2J/Kat2J mice were immunostained with anti-NEK1 and anti-CD31. Bar = 50 µm. d, Ventral horns in spinal cord lumbar sections of Nek1+/+ and Nek1Kat2J/Kat2J mice were immunostained with anti-IBA1 and anti-NEK1. Bar = 20 µm. e, H&E staining of kidney sections from mice (3 weeks old) with indicated genotypes. f-g, Visual comparison of of Nek1+/+;Ripk1D138N/D138N mice and Nek1Kat2J/Kat2J; Ripk1D138N/D138N mice at 10 months of age. h, Bodyweight of mice with indicated genotypes at 2 weeks of age. n=15 for wt; n=7 for Nek1kat2J/Kat2J mice; n=18 for Nek1+/+;Ripk1D138N/D138N mice; n= 15 for Nek1Kat2J/Kat2J;Ripk1D138N/D138N mice. One-way ANOVA with Dunnett’s test. i, Bodyweight of Nek1+/+, Nek1+/+;Ripk1D138N/D138N and Nek1Kat2J/Kat2J;Ripk1D138N/D138N mice at 5 months of age. n=7 for each group. One-way ANOVA with Dunnett’s test. j, Cortical sections from Nek1+/+ mice, Nek1Kat2J/Kat2J mice, Nek1+/+;Ripk1D138N/D138N mice and Nek1Kat2J/Kat2J;Ripk1D138N/D138N mice were immunostained with anti-cleaved Caspase-3 (CC3) and anti-CD31 antibodies. Bar = 50 µm.The cleaved Caspase-3+ endothelial cells were quantified with ImageJ (bottom). Data are represented as mean ± SD, n=6 sections for each group. One-way ANOVA with Dunnett’s test.

## Slide 3
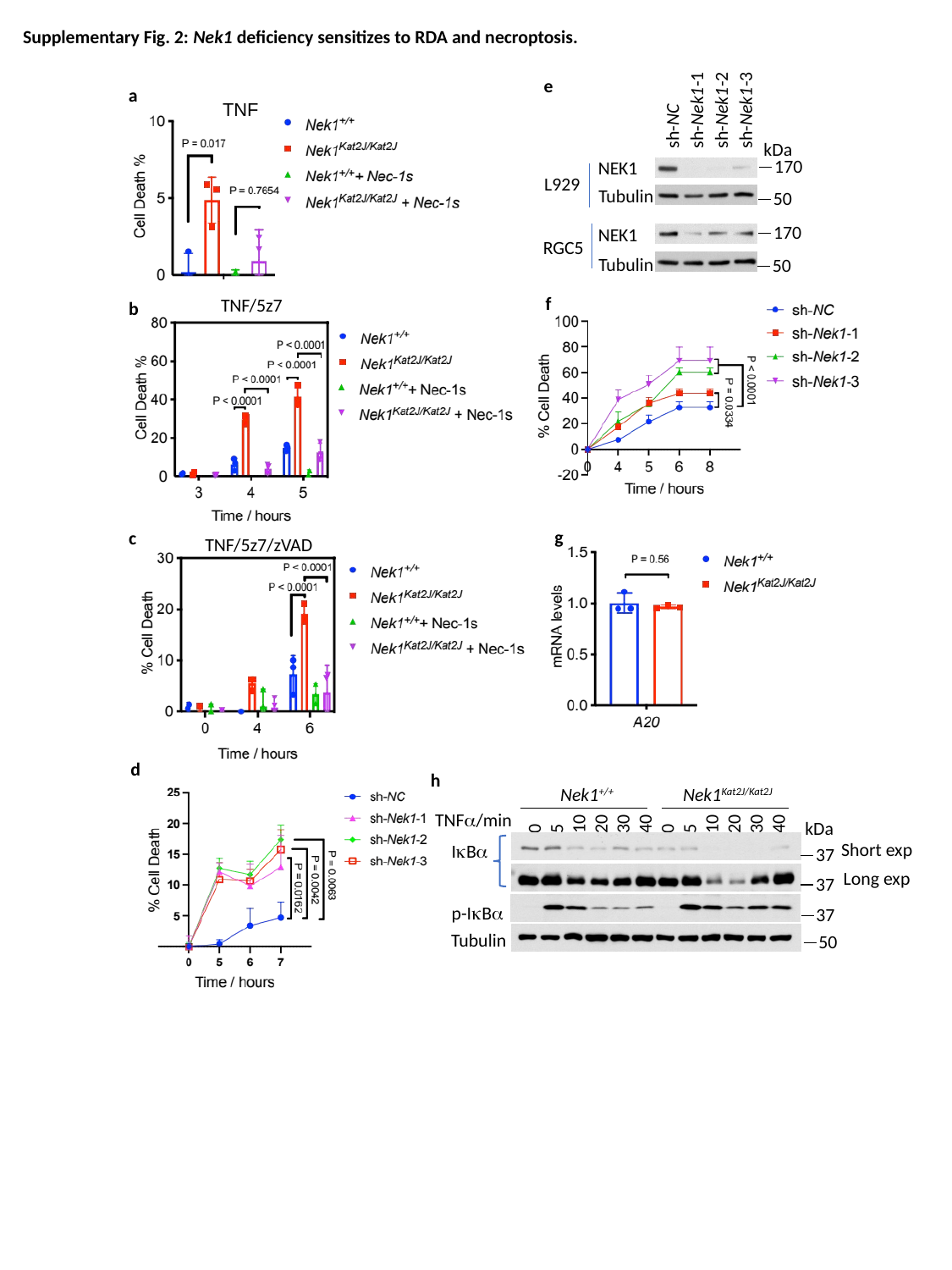

Supplementary Fig. 2: Nek1 deficiency sensitizes to RDA and necroptosis.
sh-NC
sh-Nek1-1
sh-Nek1-2
sh-Nek1-3
e
a
TNF
kDa
170
NEK1
L929
Tubulin
50
170
NEK1
RGC5
Tubulin
50
f
TNF/5z7
b
g
c
TNF/5z7/zVAD
d
0
5
10
20
30
40
0
5
10
20
30
40
h
Nek1+/+
Nek1Kat2J/Kat2J
TNFa/min
Short exp
IkBa
Long exp
p-IkBa
Tubulin
kDa
37
37
37
50

## Slide 4
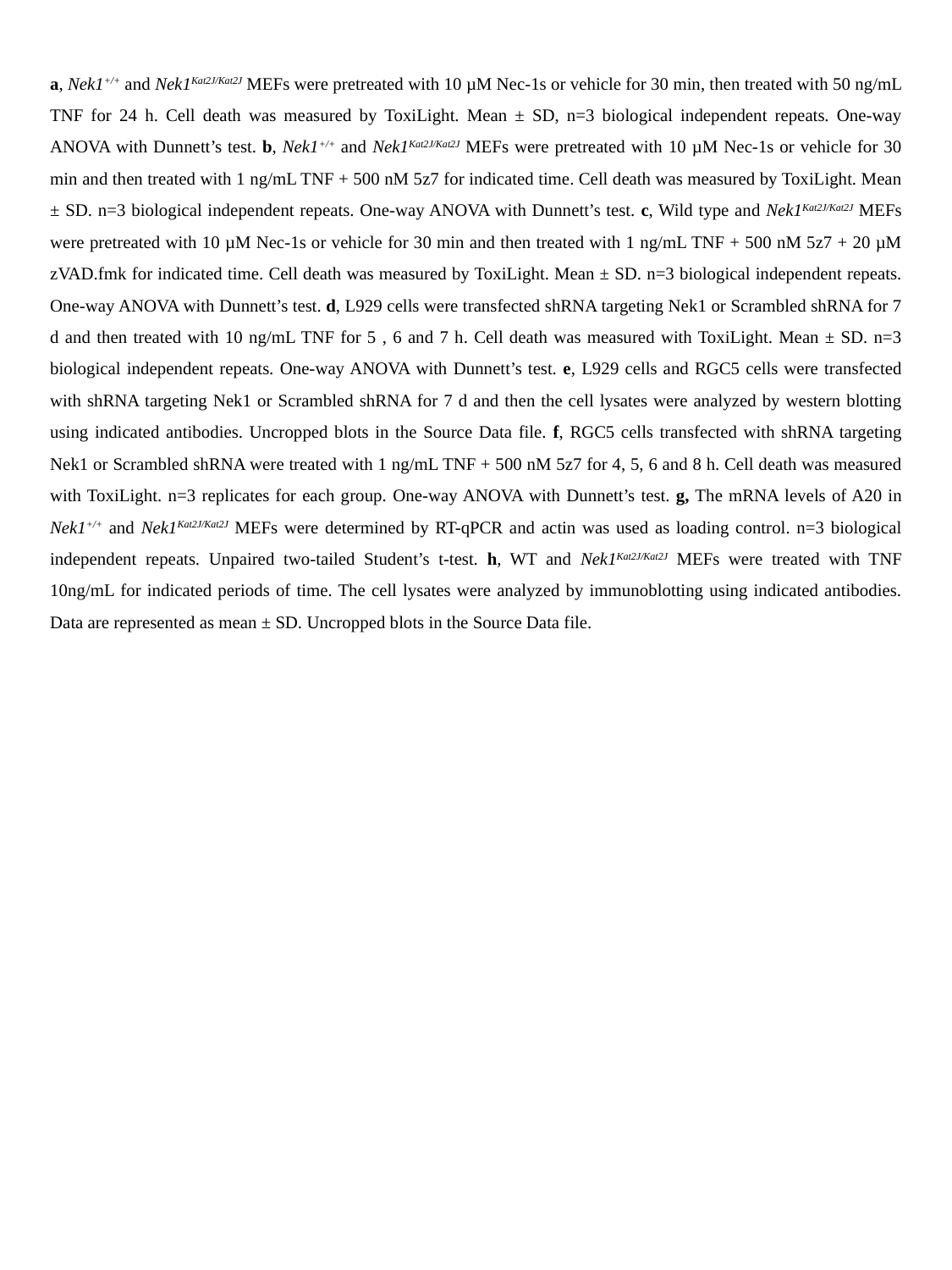

a, Nek1+/+ and Nek1Kat2J/Kat2J MEFs were pretreated with 10 µM Nec-1s or vehicle for 30 min, then treated with 50 ng/mL TNF for 24 h. Cell death was measured by ToxiLight. Mean ± SD, n=3 biological independent repeats. One-way ANOVA with Dunnett’s test. b, Nek1+/+ and Nek1Kat2J/Kat2J MEFs were pretreated with 10 µM Nec-1s or vehicle for 30 min and then treated with 1 ng/mL TNF + 500 nM 5z7 for indicated time. Cell death was measured by ToxiLight. Mean ± SD. n=3 biological independent repeats. One-way ANOVA with Dunnett’s test. c, Wild type and Nek1Kat2J/Kat2J MEFs were pretreated with 10 µM Nec-1s or vehicle for 30 min and then treated with 1 ng/mL TNF + 500 nM 5z7 + 20 µM zVAD.fmk for indicated time. Cell death was measured by ToxiLight. Mean ± SD. n=3 biological independent repeats. One-way ANOVA with Dunnett’s test. d, L929 cells were transfected shRNA targeting Nek1 or Scrambled shRNA for 7 d and then treated with 10 ng/mL TNF for 5 , 6 and 7 h. Cell death was measured with ToxiLight. Mean ± SD. n=3 biological independent repeats. One-way ANOVA with Dunnett’s test. e, L929 cells and RGC5 cells were transfected with shRNA targeting Nek1 or Scrambled shRNA for 7 d and then the cell lysates were analyzed by western blotting using indicated antibodies. Uncropped blots in the Source Data file. f, RGC5 cells transfected with shRNA targeting Nek1 or Scrambled shRNA were treated with 1 ng/mL TNF + 500 nM 5z7 for 4, 5, 6 and 8 h. Cell death was measured with ToxiLight. n=3 replicates for each group. One-way ANOVA with Dunnett’s test. g, The mRNA levels of A20 in Nek1+/+ and Nek1Kat2J/Kat2J MEFs were determined by RT-qPCR and actin was used as loading control. n=3 biological independent repeats. Unpaired two-tailed Student’s t-test. h, WT and Nek1Kat2J/Kat2J MEFs were treated with TNF 10ng/mL for indicated periods of time. The cell lysates were analyzed by immunoblotting using indicated antibodies. Data are represented as mean ± SD. Uncropped blots in the Source Data file.

## Slide 5
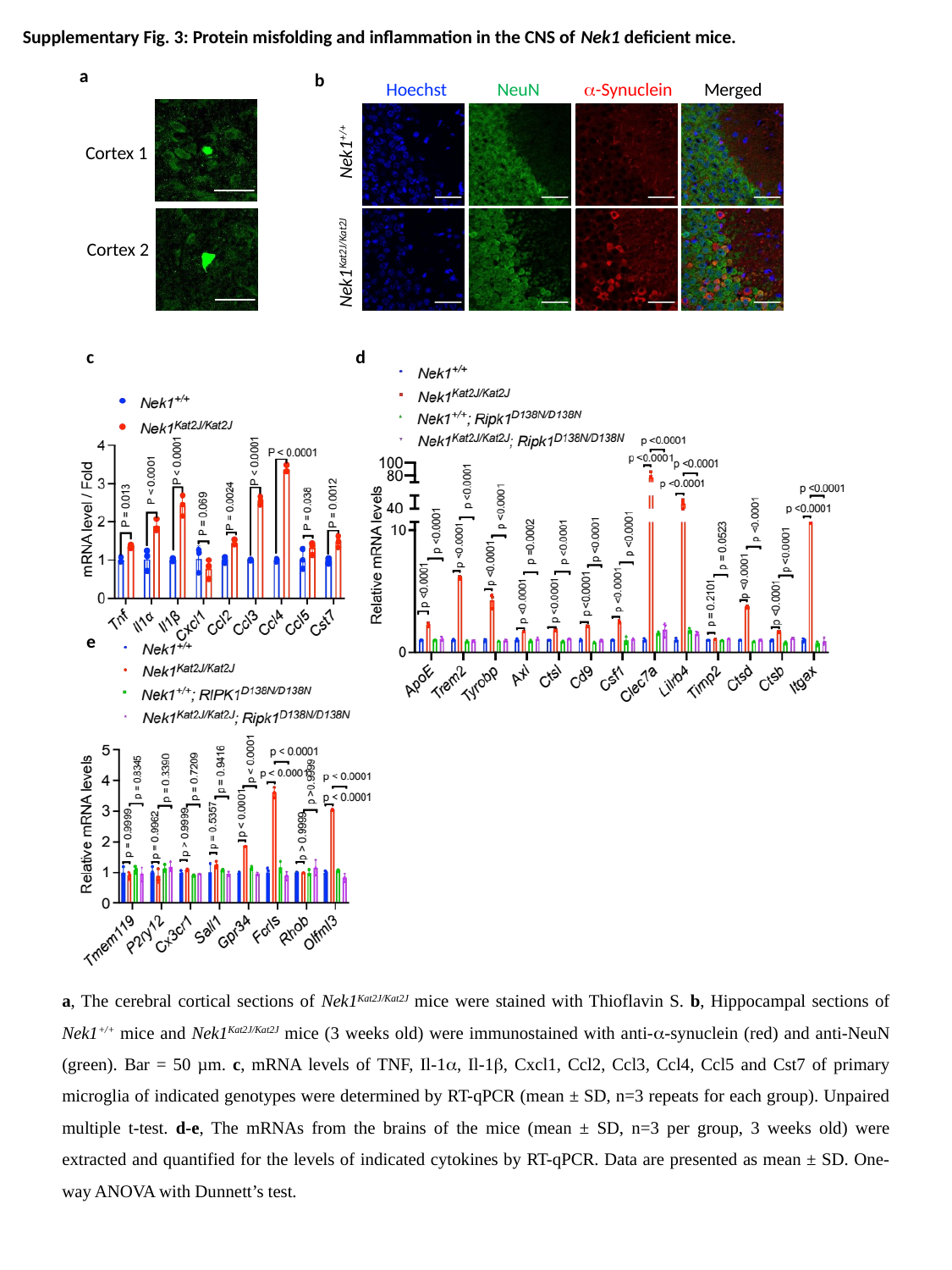

Supplementary Fig. 3: Protein misfolding and inflammation in the CNS of Nek1 deficient mice.
a
b
Hoechst
NeuN
a-Synuclein
Merged
Cortex 1
Nek1+/+
Cortex 2
Nek1Kat2J/Kat2J
c
d
e
a, The cerebral cortical sections of Nek1Kat2J/Kat2J mice were stained with Thioflavin S. b, Hippocampal sections of Nek1+/+ mice and Nek1Kat2J/Kat2J mice (3 weeks old) were immunostained with anti-a-synuclein (red) and anti-NeuN (green). Bar = 50 µm. c, mRNA levels of TNF, Il-1a, Il-1b, Cxcl1, Ccl2, Ccl3, Ccl4, Ccl5 and Cst7 of primary microglia of indicated genotypes were determined by RT-qPCR (mean ± SD, n=3 repeats for each group). Unpaired multiple t-test. d-e, The mRNAs from the brains of the mice (mean ± SD, n=3 per group, 3 weeks old) were extracted and quantified for the levels of indicated cytokines by RT-qPCR. Data are presented as mean ± SD. One-way ANOVA with Dunnett’s test.

## Slide 6
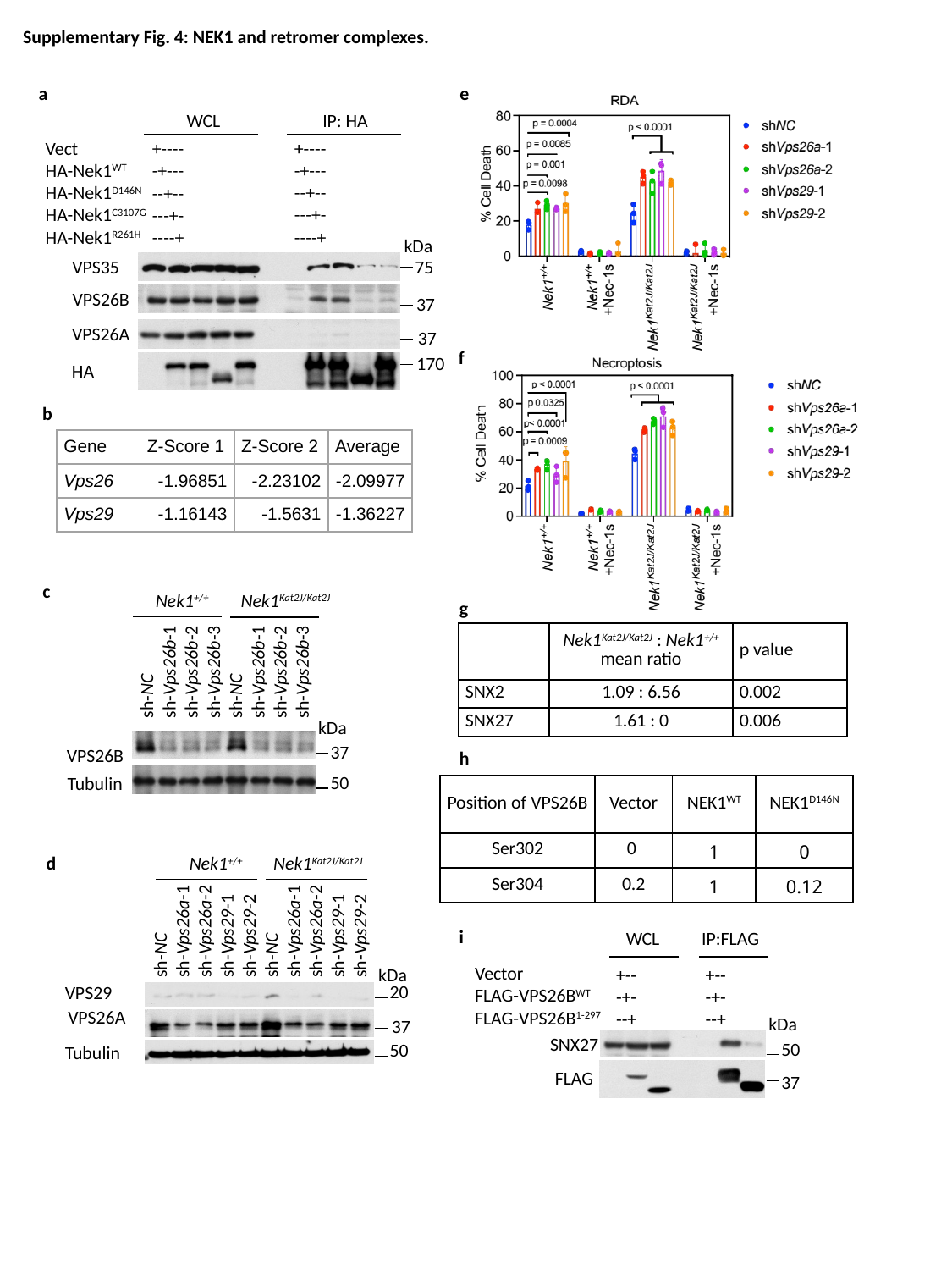

Supplementary Fig. 4: NEK1 and retromer complexes.
a
e
WCL
IP: HA
Vect
HA-Nek1WT
HA-Nek1D146N
HA-Nek1C3107G
HA-Nek1R261H
+----
-+---
--+--
---+-
----+
+----
-+---
--+--
---+-
----+
kDa
75
VPS35
VPS26B
37
VPS26A
37
f
170
HA
b
| Gene | Z-Score 1 | Z-Score 2 | Average |
| --- | --- | --- | --- |
| Vps26 | -1.96851 | -2.23102 | -2.09977 |
| Vps29 | -1.16143 | -1.5631 | -1.36227 |
c
sh-NC
sh-Vps26b-1
sh-Vps26b-2
sh-Vps26b-3
sh-NC
sh-Vps26b-1
sh-Vps26b-2
sh-Vps26b-3
Nek1+/+
Nek1Kat2J/Kat2J
g
| | Nek1Kat2J/Kat2J : Nek1+/+ mean ratio | p value |
| --- | --- | --- |
| SNX2 | 1.09 : 6.56 | 0.002 |
| SNX27 | 1.61 : 0 | 0.006 |
kDa
37
VPS26B
h
50
Tubulin
| Position of VPS26B | Vector | NEK1WT | NEK1D146N |
| --- | --- | --- | --- |
| Ser302 | 0 | 1 | 0 |
| Ser304 | 0.2 | 1 | 0.12 |
sh-NC
sh-Vps26a-1
sh-Vps26a-2
sh-Vps29-1
sh-Vps29-2
sh-NC
sh-Vps26a-1
sh-Vps26a-2
sh-Vps29-1
sh-Vps29-2
d
Nek1+/+
Nek1Kat2J/Kat2J
i
WCL
IP:FLAG
Vector
FLAG-VPS26BWT
FLAG-VPS26B1-297
kDa
+--
-+-
--+
+--
-+-
--+
20
VPS29
VPS26A
kDa
37
SNX27
50
50
Tubulin
FLAG
37

## Slide 7
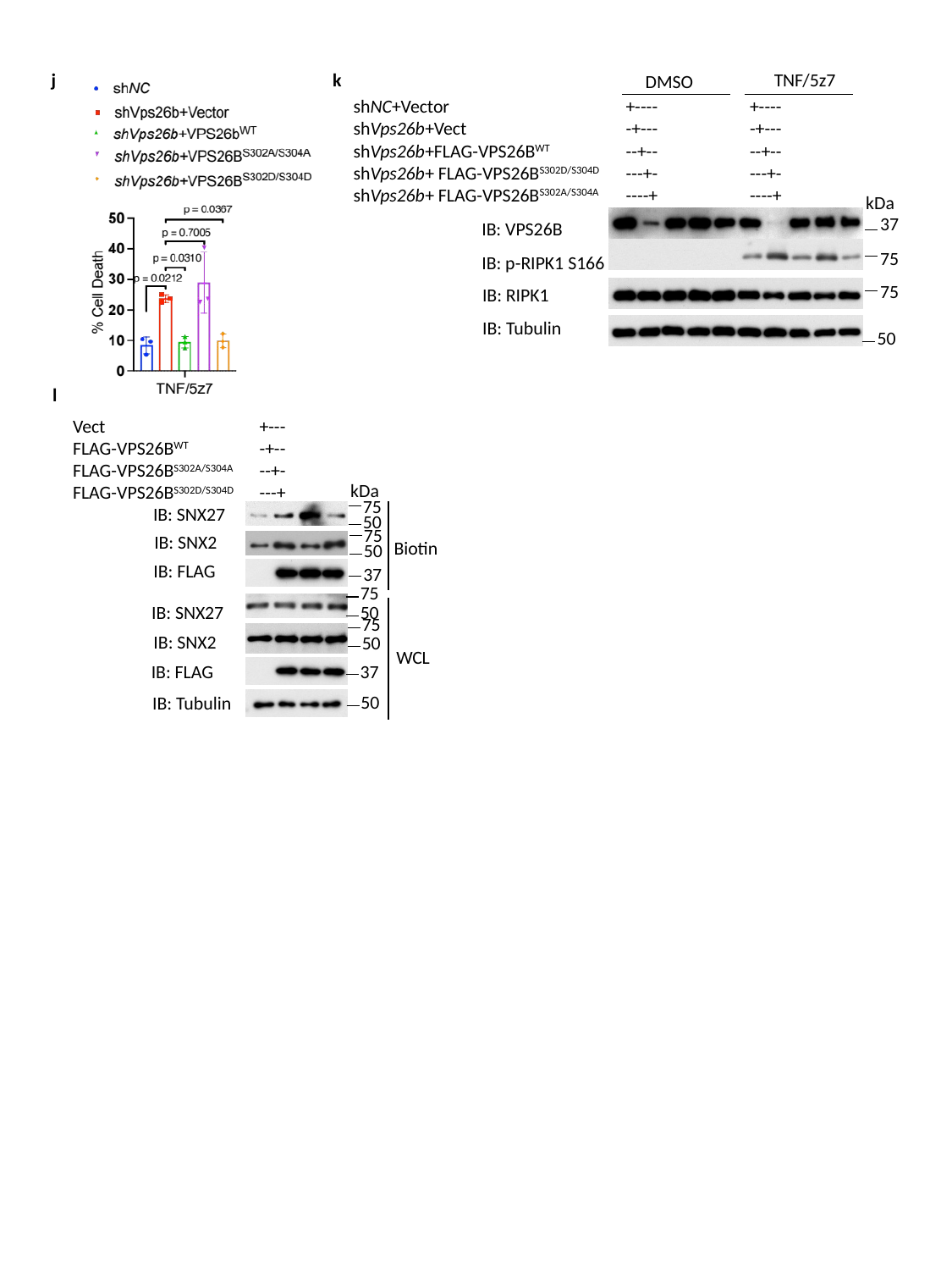

j
k
TNF/5z7
DMSO
shNC+Vector
shVps26b+Vect
shVps26b+FLAG-VPS26BWT
shVps26b+ FLAG-VPS26BS302D/S304D
shVps26b+ FLAG-VPS26BS302A/S304A
+----
-+---
--+--
---+-
----+
+----
-+---
--+--
---+-
----+
kDa
37
IB: VPS26B
75
IB: p-RIPK1 S166
75
IB: RIPK1
IB: Tubulin
50
l
+---
-+--
--+-
---+
Vect
FLAG-VPS26BWT
FLAG-VPS26BS302A/S304A
FLAG-VPS26BS302D/S304D
kDa
75
IB: SNX27
50
75
IB: SNX2
Biotin
50
IB: FLAG
37
75
50
IB: SNX27
75
IB: SNX2
50
WCL
IB: FLAG
37
50
IB: Tubulin

## Slide 8
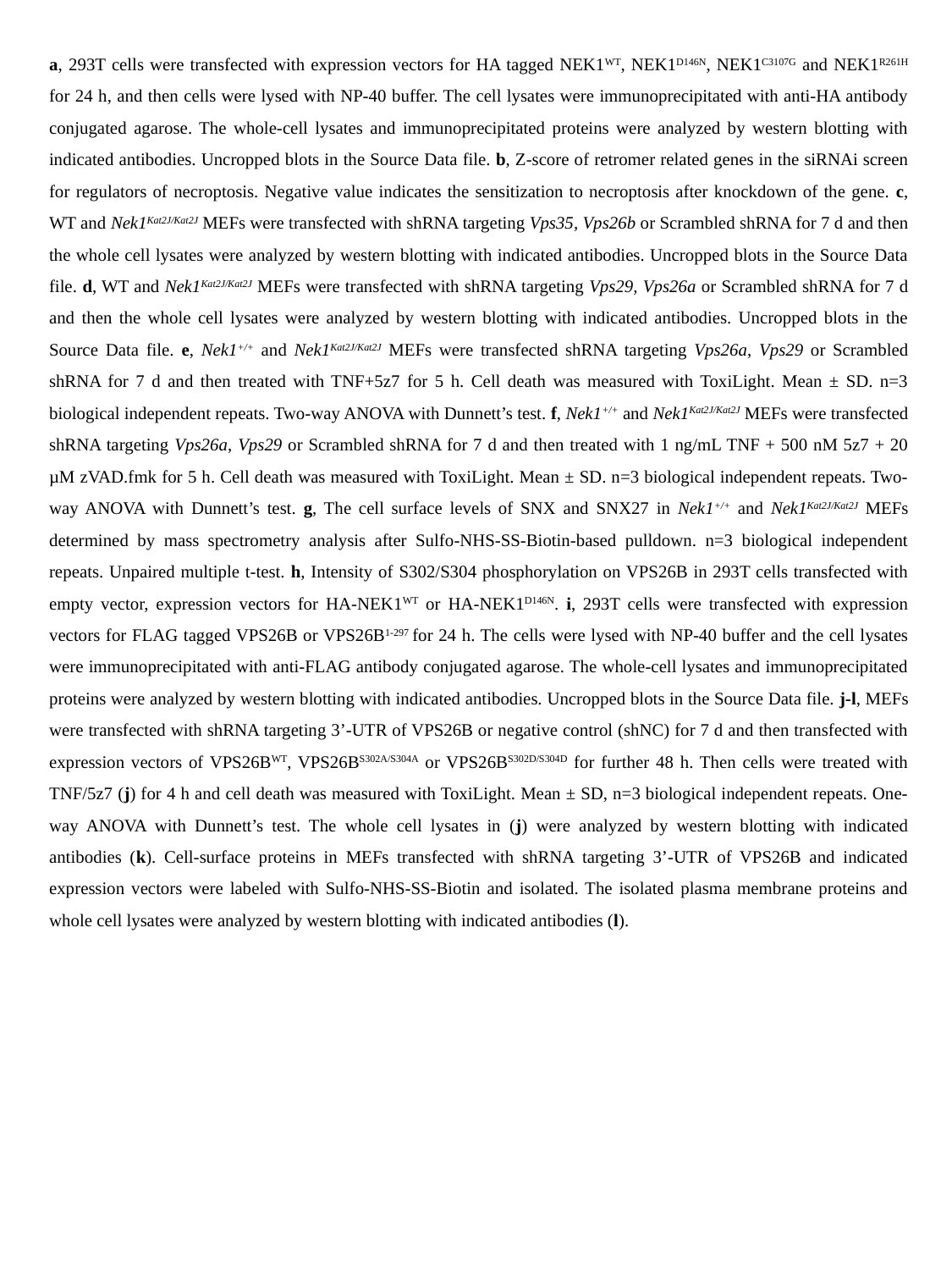

a, 293T cells were transfected with expression vectors for HA tagged NEK1WT, NEK1D146N, NEK1C3107G and NEK1R261H for 24 h, and then cells were lysed with NP-40 buffer. The cell lysates were immunoprecipitated with anti-HA antibody conjugated agarose. The whole-cell lysates and immunoprecipitated proteins were analyzed by western blotting with indicated antibodies. Uncropped blots in the Source Data file. b, Z-score of retromer related genes in the siRNAi screen for regulators of necroptosis. Negative value indicates the sensitization to necroptosis after knockdown of the gene. c, WT and Nek1Kat2J/Kat2J MEFs were transfected with shRNA targeting Vps35, Vps26b or Scrambled shRNA for 7 d and then the whole cell lysates were analyzed by western blotting with indicated antibodies. Uncropped blots in the Source Data file. d, WT and Nek1Kat2J/Kat2J MEFs were transfected with shRNA targeting Vps29, Vps26a or Scrambled shRNA for 7 d and then the whole cell lysates were analyzed by western blotting with indicated antibodies. Uncropped blots in the Source Data file. e, Nek1+/+ and Nek1Kat2J/Kat2J MEFs were transfected shRNA targeting Vps26a, Vps29 or Scrambled shRNA for 7 d and then treated with TNF+5z7 for 5 h. Cell death was measured with ToxiLight. Mean ± SD. n=3 biological independent repeats. Two-way ANOVA with Dunnett’s test. f, Nek1+/+ and Nek1Kat2J/Kat2J MEFs were transfected shRNA targeting Vps26a, Vps29 or Scrambled shRNA for 7 d and then treated with 1 ng/mL TNF + 500 nM 5z7 + 20 µM zVAD.fmk for 5 h. Cell death was measured with ToxiLight. Mean ± SD. n=3 biological independent repeats. Two-way ANOVA with Dunnett’s test. g, The cell surface levels of SNX and SNX27 in Nek1+/+ and Nek1Kat2J/Kat2J MEFs determined by mass spectrometry analysis after Sulfo-NHS-SS-Biotin-based pulldown. n=3 biological independent repeats. Unpaired multiple t-test. h, Intensity of S302/S304 phosphorylation on VPS26B in 293T cells transfected with empty vector, expression vectors for HA-NEK1WT or HA-NEK1D146N. i, 293T cells were transfected with expression vectors for FLAG tagged VPS26B or VPS26B1-297 for 24 h. The cells were lysed with NP-40 buffer and the cell lysates were immunoprecipitated with anti-FLAG antibody conjugated agarose. The whole-cell lysates and immunoprecipitated proteins were analyzed by western blotting with indicated antibodies. Uncropped blots in the Source Data file. j-l, MEFs were transfected with shRNA targeting 3’-UTR of VPS26B or negative control (shNC) for 7 d and then transfected with expression vectors of VPS26BWT, VPS26BS302A/S304A or VPS26BS302D/S304D for further 48 h. Then cells were treated with TNF/5z7 (j) for 4 h and cell death was measured with ToxiLight. Mean ± SD, n=3 biological independent repeats. One-way ANOVA with Dunnett’s test. The whole cell lysates in (j) were analyzed by western blotting with indicated antibodies (k). Cell-surface proteins in MEFs transfected with shRNA targeting 3’-UTR of VPS26B and indicated expression vectors were labeled with Sulfo-NHS-SS-Biotin and isolated. The isolated plasma membrane proteins and whole cell lysates were analyzed by western blotting with indicated antibodies (l).

## Slide 9
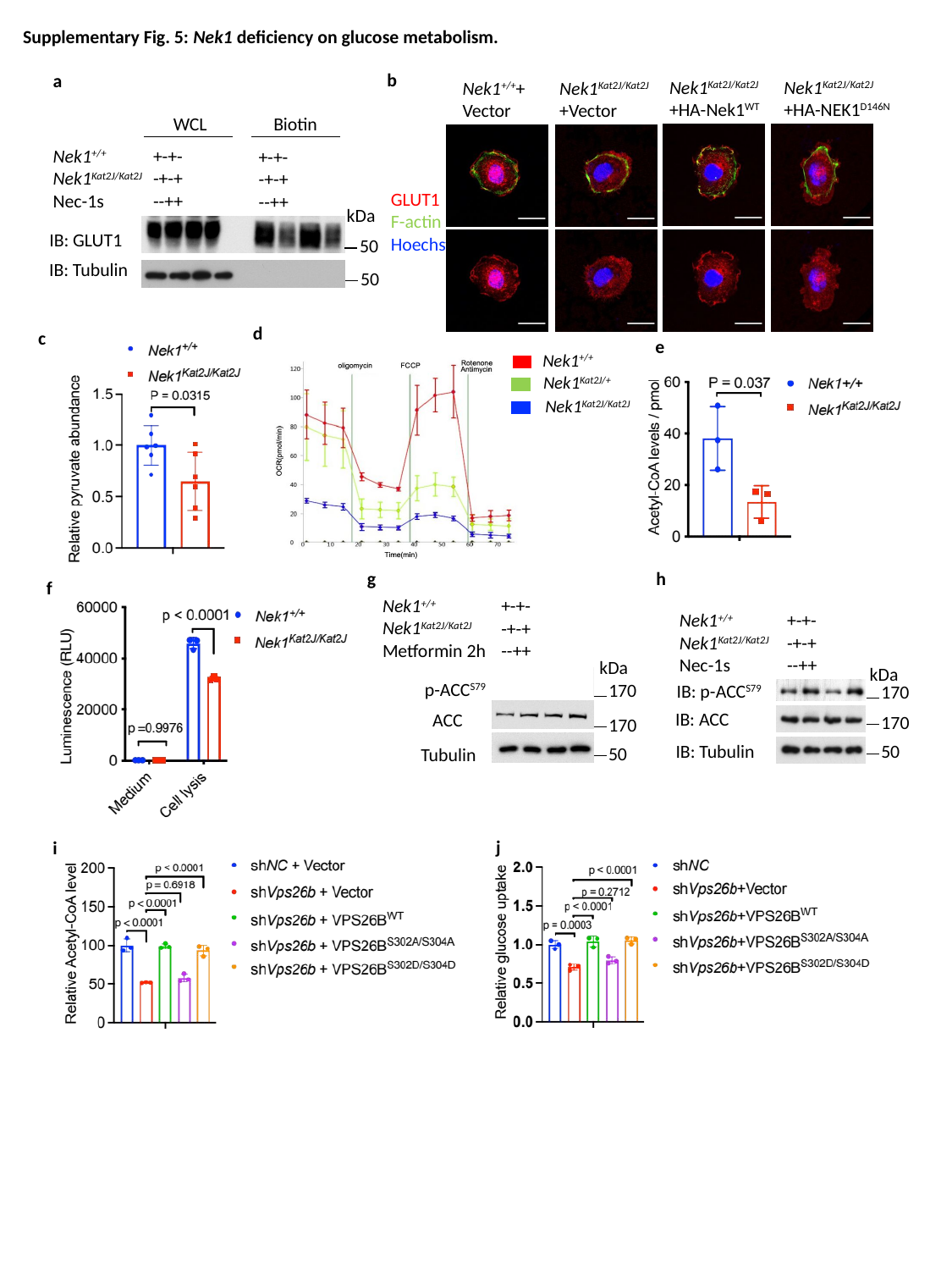

Supplementary Fig. 5: Nek1 deficiency on glucose metabolism.
b
a
Nek1Kat2J/Kat2J
+HA-Nek1WT
Nek1Kat2J/Kat2J
+HA-NEK1D146N
Nek1+/++
Vector
Nek1Kat2J/Kat2J
+Vector
WCL
Biotin
Nek1+/+
Nek1Kat2J/Kat2J
Nec-1s
+-+-
-+-+
--++
+-+-
-+-+
--++
GLUT1
F-actin
Hoechst
kDa
IB: GLUT1
50
IB: Tubulin
50
d
c
e
Nek1+/+
Nek1Kat2J/+
Nek1Kat2J/Kat2J
g
h
f
Nek1+/+
Nek1Kat2J/Kat2J
Metformin 2h
+-+-
-+-+
--++
Nek1+/+
Nek1Kat2J/Kat2J
Nec-1s
+-+-
-+-+
--++
kDa
kDa
p-ACCS79
170
IB: p-ACCS79
170
ACC
IB: ACC
170
170
Tubulin
IB: Tubulin
50
50
j
i

## Slide 10
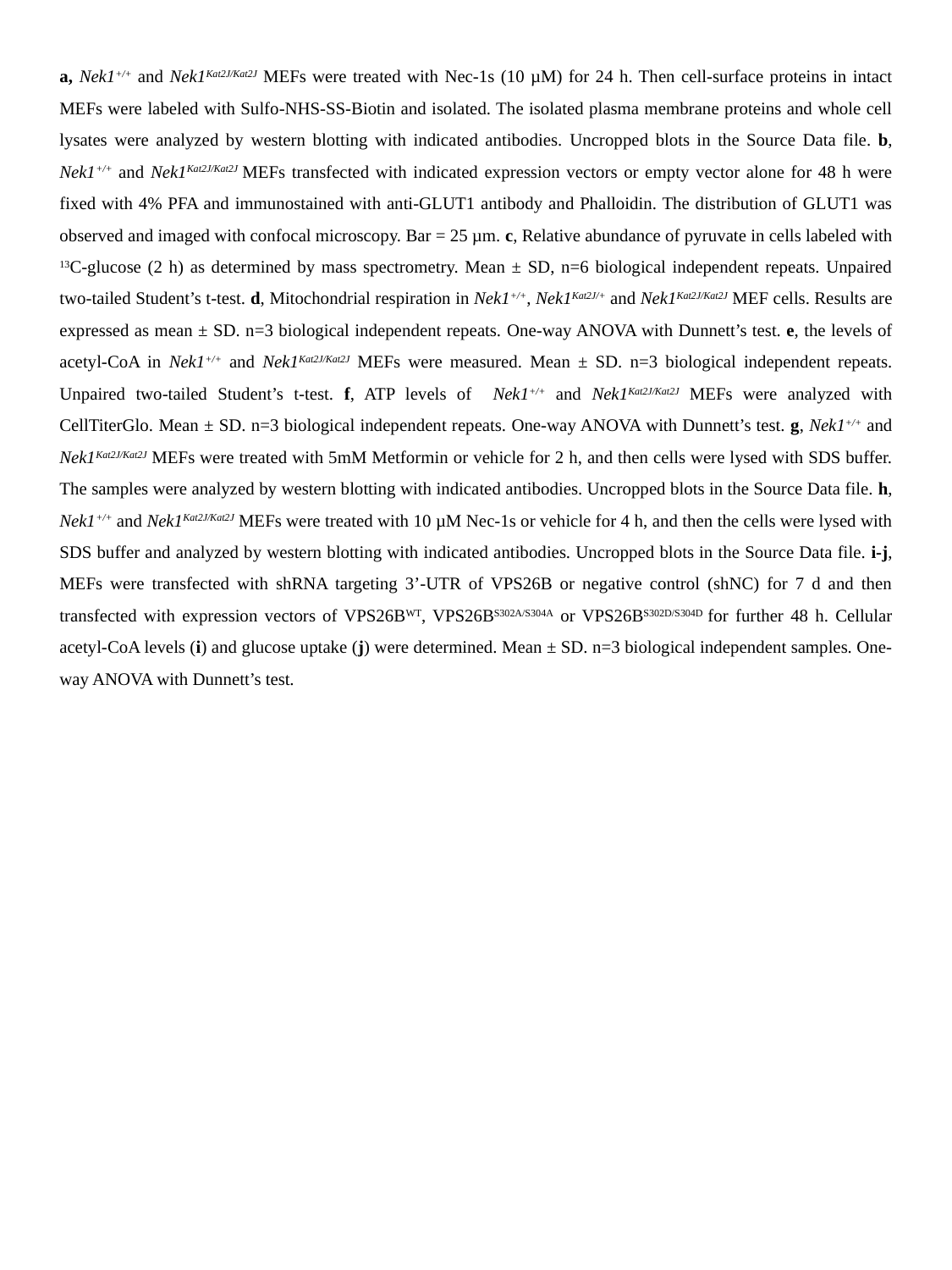

a, Nek1+/+ and Nek1Kat2J/Kat2J MEFs were treated with Nec-1s (10 µM) for 24 h. Then cell-surface proteins in intact MEFs were labeled with Sulfo-NHS-SS-Biotin and isolated. The isolated plasma membrane proteins and whole cell lysates were analyzed by western blotting with indicated antibodies. Uncropped blots in the Source Data file. b, Nek1+/+ and Nek1Kat2J/Kat2J MEFs transfected with indicated expression vectors or empty vector alone for 48 h were fixed with 4% PFA and immunostained with anti-GLUT1 antibody and Phalloidin. The distribution of GLUT1 was observed and imaged with confocal microscopy. Bar = 25 µm. c, Relative abundance of pyruvate in cells labeled with 13C-glucose (2 h) as determined by mass spectrometry. Mean ± SD, n=6 biological independent repeats. Unpaired two-tailed Student’s t-test. d, Mitochondrial respiration in Nek1+/+, Nek1Kat2J/+ and Nek1Kat2J/Kat2J MEF cells. Results are expressed as mean ± SD. n=3 biological independent repeats. One-way ANOVA with Dunnett’s test. e, the levels of acetyl-CoA in Nek1+/+ and Nek1Kat2J/Kat2J MEFs were measured. Mean ± SD. n=3 biological independent repeats. Unpaired two-tailed Student’s t-test. f, ATP levels of Nek1+/+ and Nek1Kat2J/Kat2J MEFs were analyzed with CellTiterGlo. Mean ± SD. n=3 biological independent repeats. One-way ANOVA with Dunnett’s test. g, Nek1+/+ and Nek1Kat2J/Kat2J MEFs were treated with 5mM Metformin or vehicle for 2 h, and then cells were lysed with SDS buffer. The samples were analyzed by western blotting with indicated antibodies. Uncropped blots in the Source Data file. h, Nek1+/+ and Nek1Kat2J/Kat2J MEFs were treated with 10 µM Nec-1s or vehicle for 4 h, and then the cells were lysed with SDS buffer and analyzed by western blotting with indicated antibodies. Uncropped blots in the Source Data file. i-j, MEFs were transfected with shRNA targeting 3’-UTR of VPS26B or negative control (shNC) for 7 d and then transfected with expression vectors of VPS26BWT, VPS26BS302A/S304A or VPS26BS302D/S304D for further 48 h. Cellular acetyl-CoA levels (i) and glucose uptake (j) were determined. Mean ± SD. n=3 biological independent samples. One-way ANOVA with Dunnett’s test.

## Slide 11
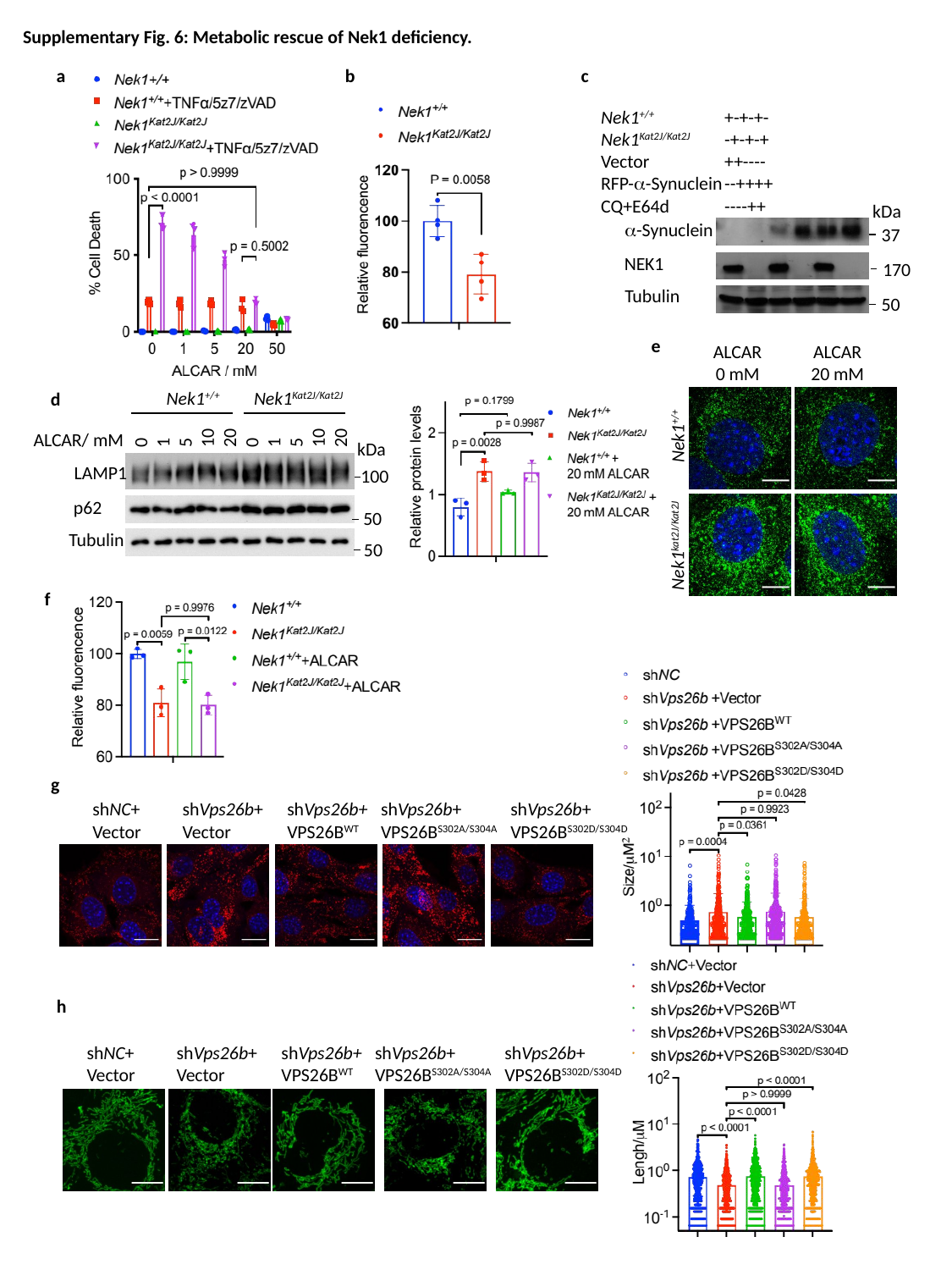

Supplementary Fig. 6: Metabolic rescue of Nek1 deficiency.
a
b
c
Nek1+/+
Nek1Kat2J/Kat2J
Vector
RFP-a-Synuclein
CQ+E64d
+-+-+-
-+-+-+
++----
--++++
----++
kDa
a-Synuclein
37
NEK1
170
Tubulin
50
0
1
5
10
20
0
1
5
10
20
e
ALCAR
0 mM
ALCAR
20 mM
Nek1Kat2J/Kat2J
Nek1+/+
d
Nek1+/+
ALCAR/ mM
kDa
LAMP1
100
p62
50
Tubulin
Nek1kat2J/Kat2J
50
f
g
shNC+
Vector
shVps26b+
Vector
shVps26b+
VPS26BWT
shVps26b+
VPS26BS302A/S304A
shVps26b+
VPS26BS302D/S304D
h
shNC+
Vector
shVps26b+
Vector
shVps26b+
VPS26BWT
shVps26b+
VPS26BS302A/S304A
shVps26b+
VPS26BS302D/S304D

## Slide 12
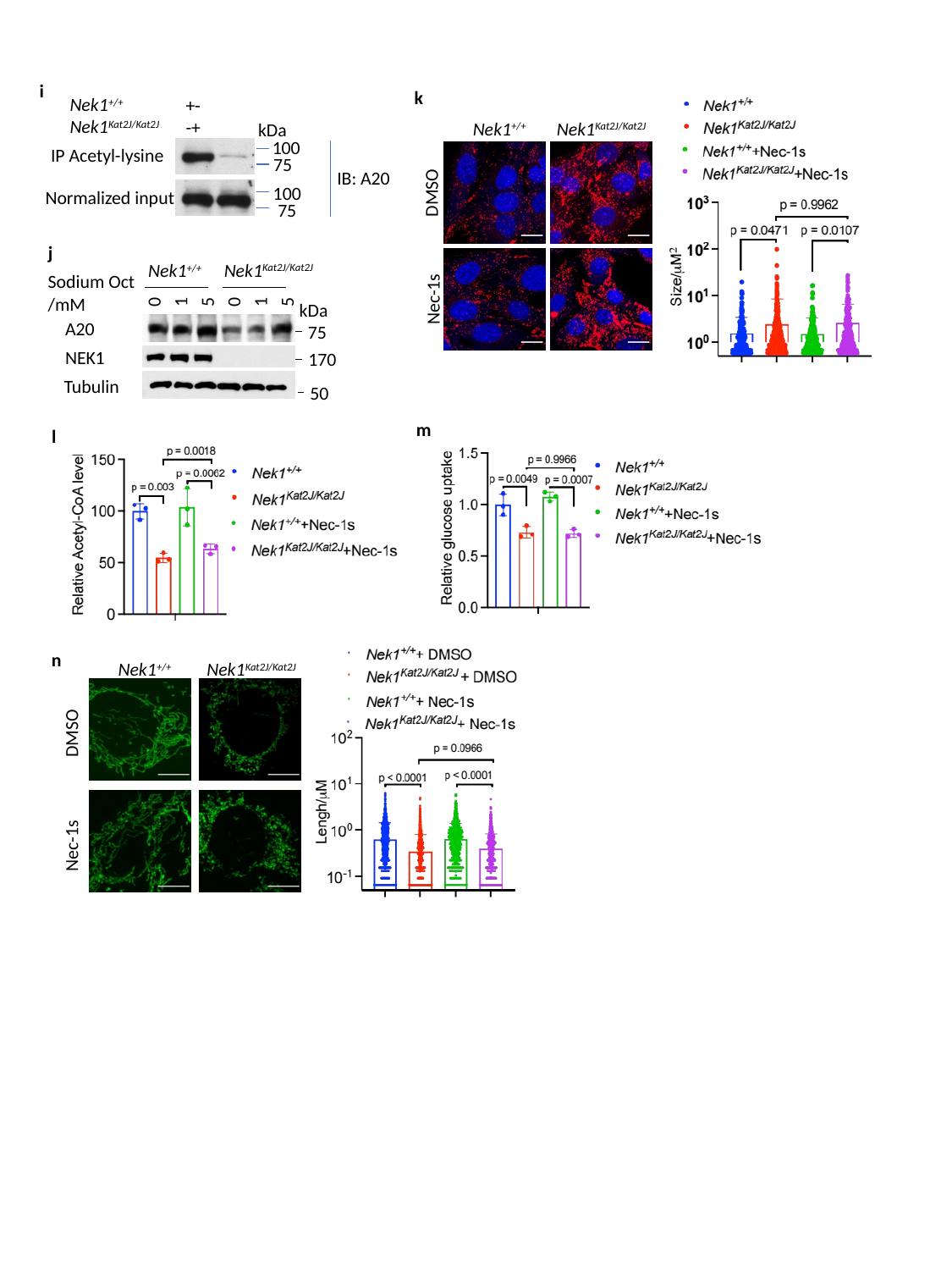

i
k
Nek1+/+
Nek1Kat2J/Kat2J
+-
-+
Nek1+/+
Nek1Kat2J/Kat2J
kDa
100
IP Acetyl-lysine
75
IB: A20
DMSO
100
Normalized input
75
j
0
1
5
0
1
5
Nek1Kat2J/Kat2J
Nek1+/+
Sodium Oct
/mM
A20
NEK1
Tubulin
Nec-1s
kDa
75
170
50
m
l
n
Nek1+/+
Nek1Kat2J/Kat2J
DMSO
Nec-1s

## Slide 13
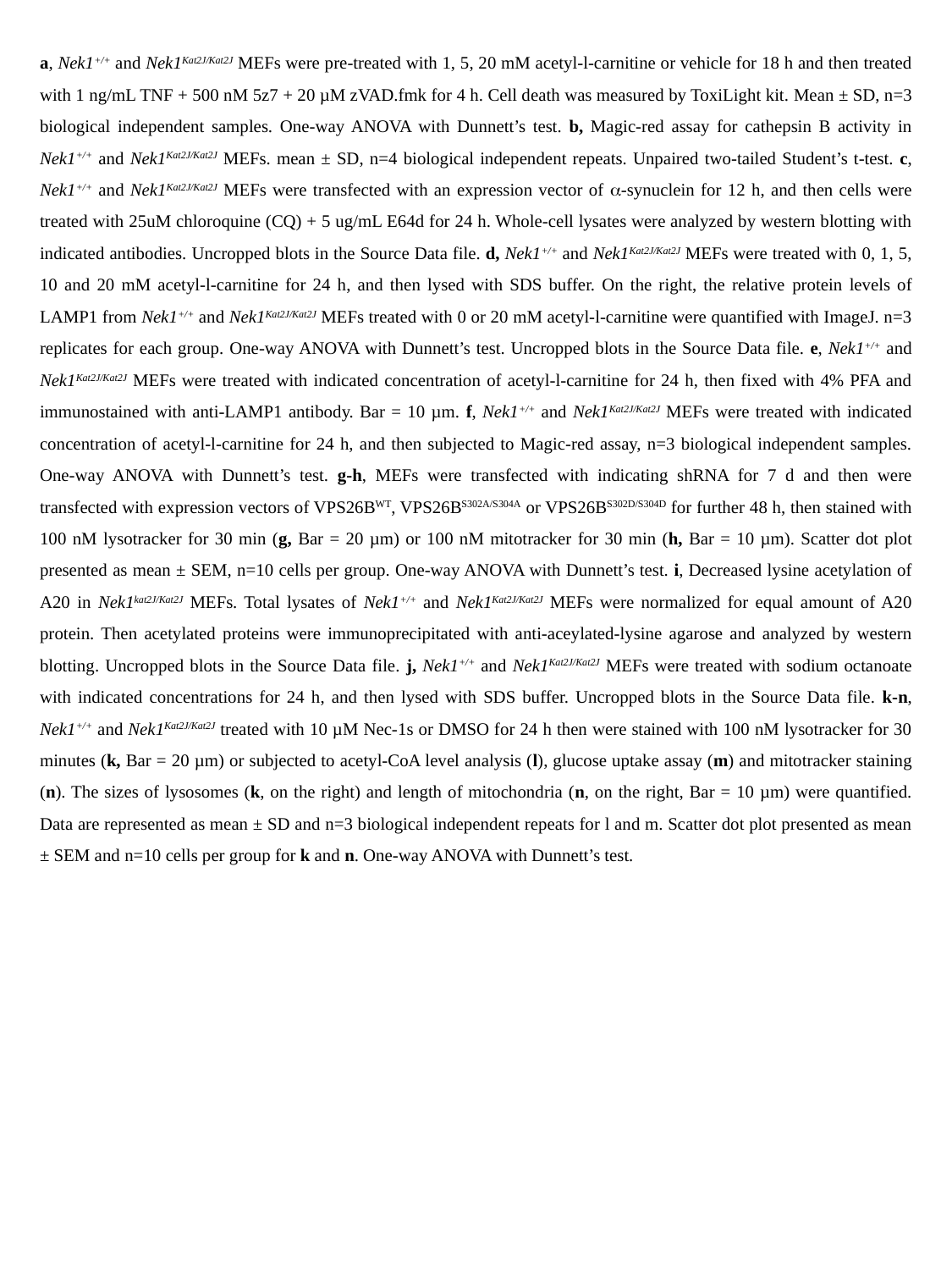

a, Nek1+/+ and Nek1Kat2J/Kat2J MEFs were pre-treated with 1, 5, 20 mM acetyl-l-carnitine or vehicle for 18 h and then treated with 1 ng/mL TNF + 500 nM 5z7 + 20 µM zVAD.fmk for 4 h. Cell death was measured by ToxiLight kit. Mean ± SD, n=3 biological independent samples. One-way ANOVA with Dunnett’s test. b, Magic-red assay for cathepsin B activity in Nek1+/+ and Nek1Kat2J/Kat2J MEFs. mean ± SD, n=4 biological independent repeats. Unpaired two-tailed Student’s t-test. c, Nek1+/+ and Nek1Kat2J/Kat2J MEFs were transfected with an expression vector of a-synuclein for 12 h, and then cells were treated with 25uM chloroquine (CQ) + 5 ug/mL E64d for 24 h. Whole-cell lysates were analyzed by western blotting with indicated antibodies. Uncropped blots in the Source Data file. d, Nek1+/+ and Nek1Kat2J/Kat2J MEFs were treated with 0, 1, 5, 10 and 20 mM acetyl-l-carnitine for 24 h, and then lysed with SDS buffer. On the right, the relative protein levels of LAMP1 from Nek1+/+ and Nek1Kat2J/Kat2J MEFs treated with 0 or 20 mM acetyl-l-carnitine were quantified with ImageJ. n=3 replicates for each group. One-way ANOVA with Dunnett’s test. Uncropped blots in the Source Data file. e, Nek1+/+ and Nek1Kat2J/Kat2J MEFs were treated with indicated concentration of acetyl-l-carnitine for 24 h, then fixed with 4% PFA and immunostained with anti-LAMP1 antibody. Bar = 10 µm. f, Nek1+/+ and Nek1Kat2J/Kat2J MEFs were treated with indicated concentration of acetyl-l-carnitine for 24 h, and then subjected to Magic-red assay, n=3 biological independent samples. One-way ANOVA with Dunnett’s test. g-h, MEFs were transfected with indicating shRNA for 7 d and then were transfected with expression vectors of VPS26BWT, VPS26BS302A/S304A or VPS26BS302D/S304D for further 48 h, then stained with 100 nM lysotracker for 30 min (g, Bar = 20 µm) or 100 nM mitotracker for 30 min (h, Bar = 10 µm). Scatter dot plot presented as mean ± SEM, n=10 cells per group. One-way ANOVA with Dunnett’s test. i, Decreased lysine acetylation of A20 in Nek1kat2J/Kat2J MEFs. Total lysates of Nek1+/+ and Nek1Kat2J/Kat2J MEFs were normalized for equal amount of A20 protein. Then acetylated proteins were immunoprecipitated with anti-aceylated-lysine agarose and analyzed by western blotting. Uncropped blots in the Source Data file. j, Nek1+/+ and Nek1Kat2J/Kat2J MEFs were treated with sodium octanoate with indicated concentrations for 24 h, and then lysed with SDS buffer. Uncropped blots in the Source Data file. k-n, Nek1+/+ and Nek1Kat2J/Kat2J treated with 10 µM Nec-1s or DMSO for 24 h then were stained with 100 nM lysotracker for 30 minutes (k, Bar = 20 µm) or subjected to acetyl-CoA level analysis (l), glucose uptake assay (m) and mitotracker staining (n). The sizes of lysosomes (k, on the right) and length of mitochondria (n, on the right, Bar = 10 µm) were quantified. Data are represented as mean ± SD and n=3 biological independent repeats for l and m. Scatter dot plot presented as mean ± SEM and n=10 cells per group for k and n. One-way ANOVA with Dunnett’s test.

## Slide 14
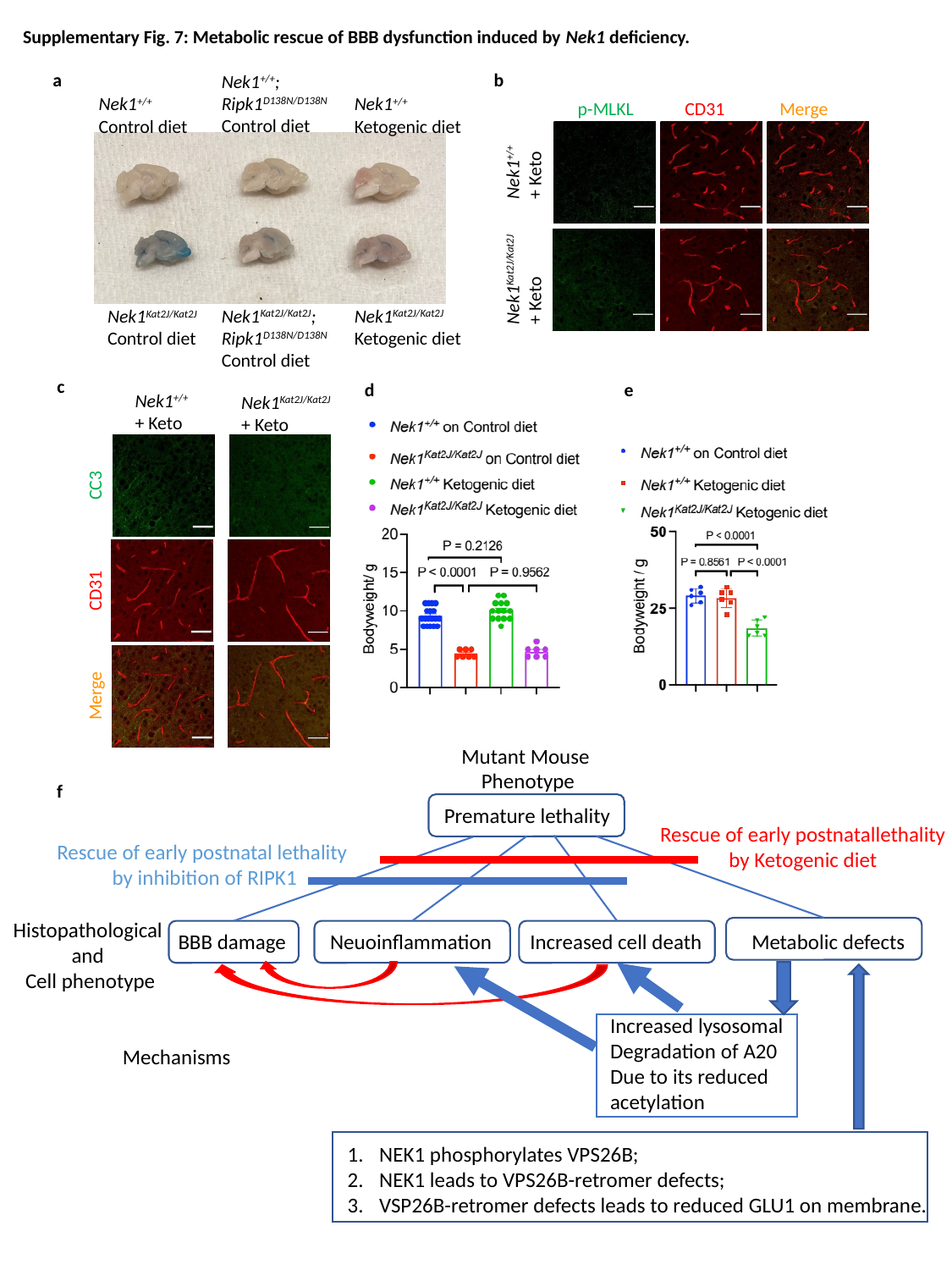

Supplementary Fig. 7: Metabolic rescue of BBB dysfunction induced by Nek1 deficiency.
a
b
Nek1+/+;
Ripk1D138N/D138N
Control diet
Nek1+/+
Control diet
Nek1+/+
Ketogenic diet
p-MLKL
CD31
Merge
Nek1+/+
+ Keto
Nek1Kat2J/Kat2J
+ Keto
Nek1Kat2J/Kat2J;
Ripk1D138N/D138N
Control diet
Nek1Kat2J/Kat2J
Ketogenic diet
Nek1Kat2J/Kat2J
Control diet
c
e
d
Nek1+/+
+ Keto
Nek1Kat2J/Kat2J
+ Keto
CC3
CD31
Merge
Mutant Mouse
 Phenotype
f
Premature lethality
Rescue of early postnatallethality
by Ketogenic diet
Rescue of early postnatal lethality
by inhibition of RIPK1
Histopathological
and
Cell phenotype
BBB damage
Neuoinflammation
Increased cell death
Metabolic defects
Increased lysosomal
Degradation of A20
Due to its reduced
acetylation
Mechanisms
NEK1 phosphorylates VPS26B;
NEK1 leads to VPS26B-retromer defects;
VSP26B-retromer defects leads to reduced GLU1 on membrane.

## Slide 15
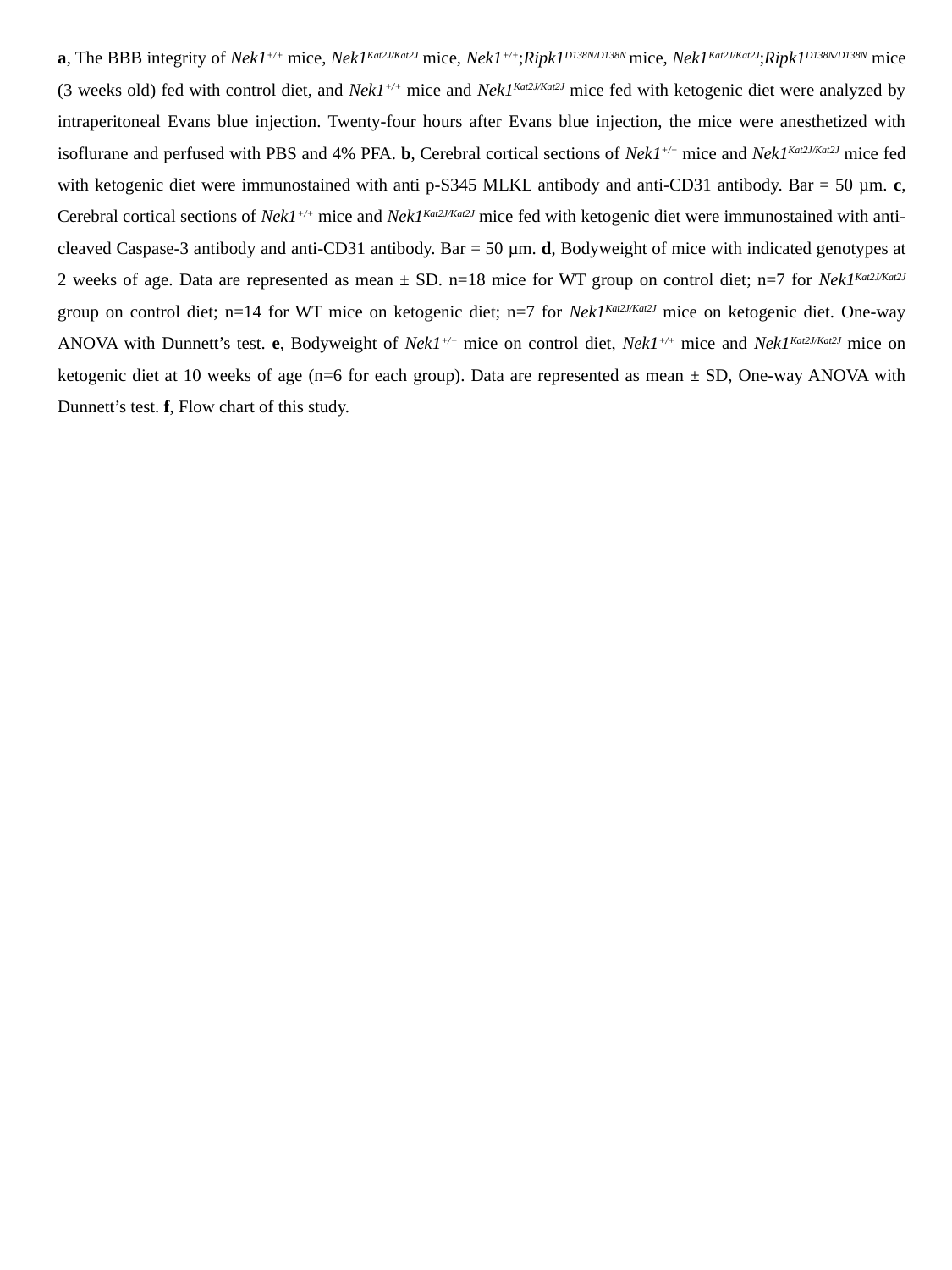

a, The BBB integrity of Nek1+/+ mice, Nek1Kat2J/Kat2J mice, Nek1+/+;Ripk1D138N/D138N mice, Nek1Kat2J/Kat2J;Ripk1D138N/D138N mice (3 weeks old) fed with control diet, and Nek1+/+ mice and Nek1Kat2J/Kat2J mice fed with ketogenic diet were analyzed by intraperitoneal Evans blue injection. Twenty-four hours after Evans blue injection, the mice were anesthetized with isoflurane and perfused with PBS and 4% PFA. b, Cerebral cortical sections of Nek1+/+ mice and Nek1Kat2J/Kat2J mice fed with ketogenic diet were immunostained with anti p-S345 MLKL antibody and anti-CD31 antibody. Bar = 50 µm. c, Cerebral cortical sections of Nek1+/+ mice and Nek1Kat2J/Kat2J mice fed with ketogenic diet were immunostained with anti-cleaved Caspase-3 antibody and anti-CD31 antibody. Bar = 50 µm. d, Bodyweight of mice with indicated genotypes at 2 weeks of age. Data are represented as mean ± SD. n=18 mice for WT group on control diet; n=7 for Nek1Kat2J/Kat2J group on control diet; n=14 for WT mice on ketogenic diet; n=7 for Nek1Kat2J/Kat2J mice on ketogenic diet. One-way ANOVA with Dunnett’s test. e, Bodyweight of Nek1+/+ mice on control diet, Nek1+/+ mice and Nek1Kat2J/Kat2J mice on ketogenic diet at 10 weeks of age (n=6 for each group). Data are represented as mean ± SD, One-way ANOVA with Dunnett’s test. f, Flow chart of this study.

## Slide 16
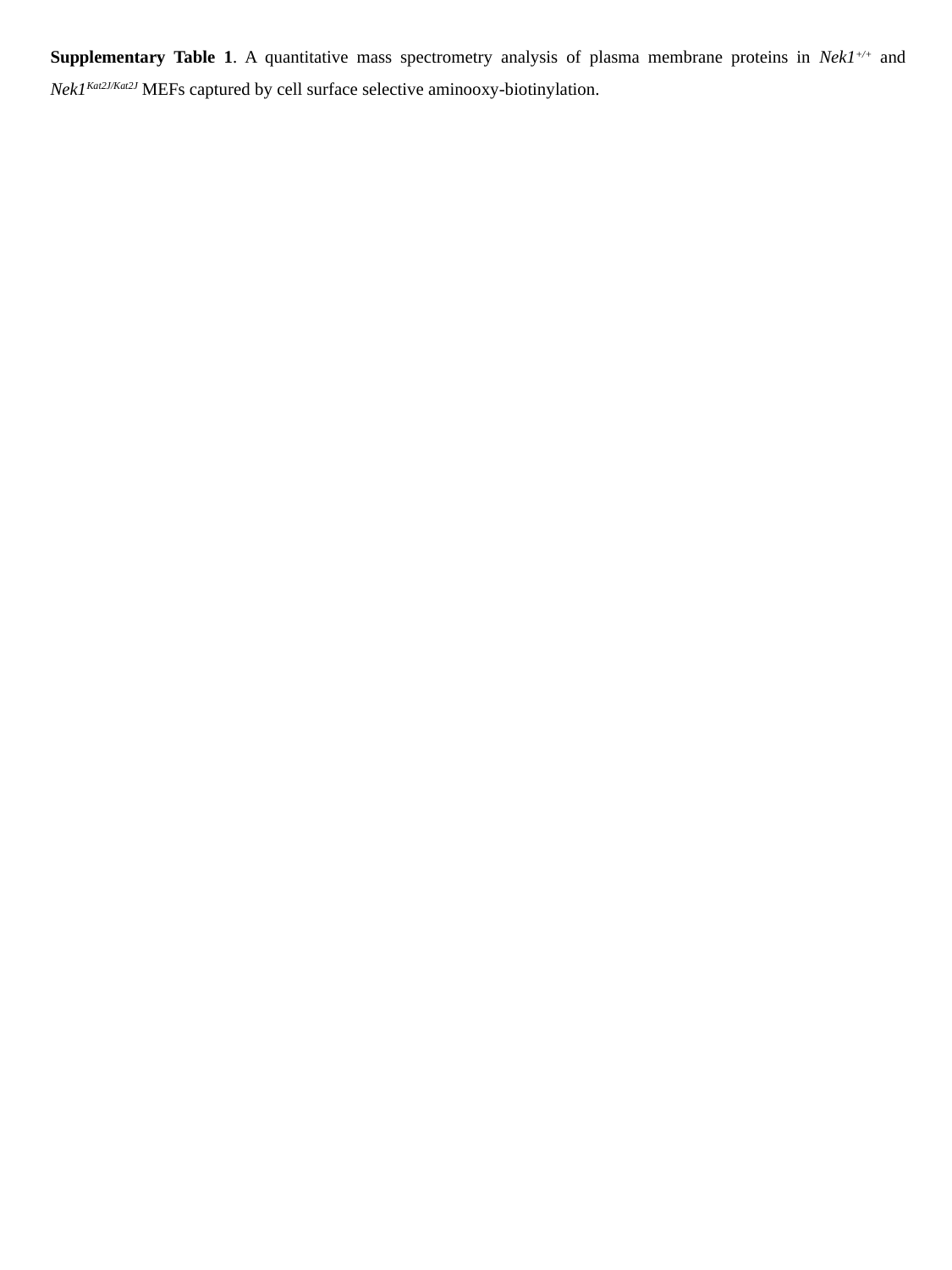

Supplementary Table 1. A quantitative mass spectrometry analysis of plasma membrane proteins in Nek1+/+ and Nek1Kat2J/Kat2J MEFs captured by cell surface selective aminooxy-biotinylation.
